# Interaction between the Bird Cherry-Oat Aphid (*Rhopalosiphum padi*) and Stagonospora Nodorum Blotch (*Parastagonospora nodorum*) on Wheat

**DOI:** 10.3390/insects12010035

**Published:** 2021-01-06

**Authors:** Belachew Asalf, Andrea Ficke, Ingeborg Klingen

**Affiliations:** Division of Biotechnology and Plant Health, Norwegian Institute of Bioeconomy Research (NIBIO), 1433 Ås, Norway; andrea.ficke@nibio.no (A.F.); ingeborg.klingen@nibio.no (I.K.)

**Keywords:** aphid, fungal plant disease, *Rhopalosiphum padi*, Parastagonospora nodorum, stagonospora nodorum blotch, wheat, plant-pathogen-herbivore interaction, phytobiome

## Abstract

**Simple Summary:**

The bird cherry-oat aphid and the fungal plant pathogen causing stagonospora nodorum blotch (SNB) are common pests of wheat. Plants are under constant attack by multiple pests and diseases but there are limited studies on the interaction between several pests on wheat. We therefore conducted controlled greenhouse and laboratory experiments to determine how these pests affected each other on a wheat plant. We found that aphid feeding predisposed wheat to fungal disease, but that aphids preferred and reproduced better on leaves that had not been infected by the fungal pathogen. These results are important to understand the interactions between multiple pests on wheat and how to develop new control strategies in future integrated pest management (IPM).

**Abstract:**

Wheat plants are under constant attack by multiple pests and diseases. Until now, there are no studies on the interaction between the aphid *Rhopalosiphum padi* and the plant pathogenic fungus *Parastagonospora nodorum* causal agent of septoria nodorum blotch (SNB) on wheat. Controlled experiments were conducted to determine: (i) The preference and reproduction of aphids on *P. nodorum* inoculated and non-inoculated wheat plants and (ii) the effect of prior aphid infestation of wheat plants on SNB development. The preference and reproduction of aphids was determined by releasing female aphids on *P. nodorum* inoculated (SNB+) and non-inoculated (SNB−) wheat leaves. The effect of prior aphid infestation of wheat plants on SNB development was determined by inoculating *P. nodorum* on aphid-infested (Aphid+) and aphid free (Aphid−) wheat plants. Higher numbers of aphids moved to and settled on the healthy (SNB−) leaves than inoculated (SNB+) leaves, and reproduction was significantly higher on SNB− leaves than on SNB+ leaves. Aphid infestation of wheat plants predisposed the plants to *P. nodorum* infection and colonization. These results are important to understand the interactions between multiple pests in wheat and hence how to develop new strategies in future integrated pest management (IPM).

## 1. Introduction

Wheat (*Triticum aestivum*) plants are often under simultaneous or sequential attack of pests from multiple unrelated groups of pests. In this paper, we will use the term pest to refer to plant pathogens and arthropods (mites and insects) as defined by [1] to be any species, strain or biotype of plant, animal, or pathogenic agent injurious to plants or plant products. The bird cherry-oat aphid (*Rhopalosiphum padi* (Aphididae: Hemiptera)) and the necrotrophic pathogen, *Parastagonospora nodorum*, the causal agent of stagonospora nodorum blotch (SNB) are economically important pests of wheat. Aphid infestation start early in the wheat growing season, whereas SNB becomes more severe late in the wheat growing season. In northern Europe, the two main aphid species in cereals are *R. padi* and the grain aphid *Sitobion avenae*. They are serious insect pests on cereals and share host plants in the Poaceae (grass) family, which includes crops like wheat [2]. Aphids damage cereals directly by sucking phloem sap, and indirectly by transmitting viruses and reducing photosynthesis by depositing honeydew that decrease photosynthesis, stimulate leaf senescence and growth of sooty mold [3]. *Rhopalosiphum padi* has a wide geographic distribution and correspondingly different life cycles [4]. Further it has an anholocyclic life cycle in cereals during the cropping season. In the beginning of the season they place themselves on the plant close to the soil surface. Then they colonize more of the plant and place themselves mainly on the underside of the leaves [5]. When winter comes, it migrates to its winter host bird cherry (*Prunus padus*) where it overwinters as eggs close to the buds on the branches. Aphids can cause yield losses as high as 40% in wheat [6] and SNB can cause up to 50% of yield loss in susceptible cultivars [7].

Microbe- or insect- induced changes of plant resistance towards greater or lesser susceptibility to the second attacker is a well-documented phenomenon in pest-plant interactions [8,9,10,11,12,13,14,15], and several mechanisms such as priming of the plant by activating the salicylic acid (SA)-pathway are suggested to be involved [16]. Aphid and plant pathogens can interact directly through competition for resources and space, and indirectly by affecting the host response either positively (induced resistance) or negatively (induced susceptibility) and by changing the microclimate, nutrition status and physiochemical condition of the host and even by affecting each other’s natural enemies [9,12,13,14].

Disease-mediated aphid-plant interactions can be positive, negative or neutral on preference and population growth of the aphid [8,13,17]. Infection of plants by necrotrophic or biotrophic fungi and pathogenic bacteria are reported to lead to either an increase or a decrease in the performance of aphids on plants [18]. For instance, *Botrytis cinerea* inhibits the black bean aphid (*Aphis fabae*) development, survival, fecundity and performance on Broad beans (*Vicia faba*) [13]. Pre-infection of rose plants (*Rosa hybrid* cv. Sonia) by *B. cinerea* reduces the yellow rose aphid (*Rhodobium porosum*) population growth [10], and pre-infection of pepper (*Capsicum annuum*) with a plant pathogenic bacterium, *Xanthomonas axonopodis* pv. *Vesicatoria*, reduced the green peach aphid (*Myzus persicae*) population [19]. In contrast, aphid performance was enhanced on Broad beans (*V. faba*) infected by *Botrytis fabae*, and it was speculated that nutrient supply to aphids increased on diseased leaves as opposed to the healthy leaves [20]. The biotrophic rust fungus (*Uromyces viciae-fabae*) is also reported to enhances aphid performance on Broad beans [13].

Similarly, insect-mediated plant pathogen–plant interactions can be positive, negative or neutral to the plant disease development [13]. There are several studies that show a negative effect of insect-induced changes in the host plant on disease development [8,12,15,21]. Pre-infestation of rose plants, *Rosa hybrid* cv. Sonia, by the yellow rose aphid *R. porosum* significantly reduces the disease severity of *Botrytis cinerea* [10]. However, in wheat, aphid infestation increased fusarium head blight severity caused by *Fusarium graminearum* 2-fold [14]. Studies on trees showed that prior infestation of conifers with the large pine aphid, *Cinara pinea*, increased the disease symptoms caused by the plant pathogenic fungus *Gremmeniella abietina* [15].

Numerous studies are available on single insect–plant interactions (aphid–cereal [6] and SNB−wheat interactions [22]). Although the two-way interaction studies between insect-host and pathogen-host are important to understand the basic infection and colonization processes and to model the epidemic development of SNB and the outbreak of aphids, it is an extreme simplification of nature’s complexity. The classical one pest and host interaction may not represent what happen under field conditions as multiple pests appear in parallel. Little is, however, done on the interaction between aphids and SNB. The objectives were therefore to (i) determine the preference and reproduction of aphids on SNB pre-inoculated and non-inoculated wheat plants, and (ii) determine the effect of prior aphid infestation on SNB development.

## 2. Materials and Methods

### 2.1. Plant Material

Spring wheat were used for the interaction studies. Plastic pots (12 cm diameter) were filled with a peat based potting compost P–Jord (70% Sphagnum peat H2–H4, 20% Sphagnum peat H6–H8, 10% sand. L.O.G. AS, Oslo, Norway) and placed on a plastic tray to allow watering from the bottom. Five seeds per pot were sown at a depth of 1–2 cm. The pots were kept in a greenhouse compartment at 22 ± 1 °C, 70% relative humidity (RH), and a 16:8 h day: night regime. High-pressure sodium (HPS) lamps provided additional daylight–balanced light whenever light intensity went below 150 μmol m^−2^ s^−1^. Fertilizer was applied with irrigation water formulated by mixing stock solutions of Superba RødTM (7-4-22 NPK+ micronutrients) and CalcinitTM (15.5% N, 19% Ca) in equal proportions until the electrical conductivity (EC) of the nutrient solution was around 1.7. After the seeds germinated, the plants were thinned down to four plants per pot.

### 2.2. Source of Aphids and P. nodorum

Bird cherry-oat aphid (*R. padi*) were used for both the interaction and choice experiments. *Rhopalosiphum padi* culture was established from a single individual collected from Bird cherry (*Prunus padus*) in 2012 in Toten, Norway (60.5536 N, 10.9309 E) and maintained on wheat plants in a climate room at 22 ± 1 °C, 50–70% RH and a 16:8 h day: night regime at NIBIO, Division of Biotechnology and Plant Health, Ås, Norway.

*Parastagonospora nodorum* was obtained from our laboratory isolate collections (isolate 201254). Pycnidiospores were produced in vegetable juice (V8) agar medium after incubation at 20 °C, 12 h near UV light 12 h darkness for 10–14 days. After sporulation, pycnidia were scraped off the agar with a plastic spatula and washed off with distilled water that contained tween 20 (0.1% *v/v*). The pycnidiospore suspension was filtered through a double layered cheese cloth and adjusted to 10^6^ mL^−1^ spores for final inoculation. The suspension was used within 1 h after preparation to ensure spore viability.

### 2.3. Experimental Set Up

#### 2.3.1. Effect of Aphid Infestation on SNB Development

To test the effect of aphid colonization on SNB development, the spring wheat cultivar ‘Bjarne’ was exposed to aphids at BBCH 37 (flag leaf visible, still rolled) by releasing two adult female aphids (*R. padi*) on the penultimate leaves of each tiller in insect-proof cages compartment inside a greenhouse. There were four treatment combinations per experiment (Table 1). The abbreviation BBCH derives from Germany words Biologische Bundesanstalt, Bundessortenamt and Chemical industry, and it is a system for a uniform coding of phenologically similar growth stages of plants.

A plant had on average about four tillers at the time of aphid release. After exposing the plants to aphid infestation for 7–10 days, aphids were removed by applying the insecticide BISCAYA, (active ingredient thiacloprid 240 g/L (22.97% *w/w*) at the recommended dose (400 mL/hectare in 200 L water)) to avoid further aphid colonization of plants and hence the destruction of plants and the experiment. Treatments that required inoculation of *P. nodorum* were then inoculated 24 h after aphid removal. This was done by spraying the spore suspension (10^6^ mL^−1^) on wheat plants (at BBCH 37) until run off using a handheld sprayer. After inoculation, plants were covered with clear plastic bags to increase RH to 100% for 48 h to ensure climate conditions conducive for SNB infection. Control (un-inoculated) plants were sprayed with water and covered with plastic bags to create a microclimate similar to the inoculated plants.

Plants of each treatment were kept in separate insect-proof cages in a greenhouse compartment at 20 °C, 70% RH and a 16:8 h light: darkness regime. The experiment was repeated three times over time as shown in Table 2 with repetition 1, 2 and 3 having three, four and five replicates per treatment, respectively. Each tiller had on average 4 leaves at time of disease registration.

Disease incidence was assessed on leaves of three arbitrarily selected tillers per plant. All the leaves per tiller were assessed for SNB symptoms, and then disease incidence (percentage of infected leaves per total numbers of leaves) was determined. Disease severity (percent leaf area infected) was assessed on penultimate leaves of each tiller and the infected leaf area, which was percentage of the leaf area covered by the disease, was estimated visually.

#### 2.3.2. Aphid Preference and Reproduction on SNB Inoculated Versus Non-Inoculated Leaves

*Parastagonospora nodorum* inoculation and inoculum production were conducted as described above. Wheat plants (at BBCH 37) were evenly sprayed with a 1 × 10^6^ mL^−1^ spore suspension of *P. nodorum* conidia until run off. The inoculated plants were covered with plastic bags for about 48 h to create a conducive climate for SNB infection. Control (*P. nodorum* un-inoculated) plants were sprayed with water and covered with plastic bags to create a microclimate similar to the inoculated plants. Two weeks after inoculation, leaves that show equal level of *P. nodorum* infection were selected from SNB inoculated (SNB+) and healthy leaves from SNB non- inoculated plants (SNB−).

**Figure 1:**
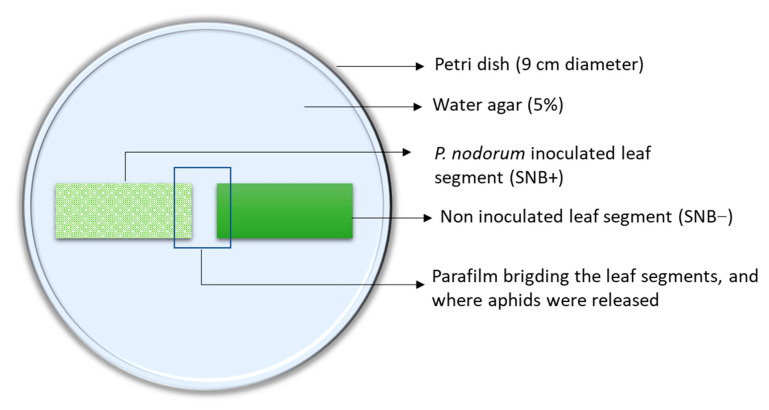
Aphid (*Rhopalosiphum padi*) choice experiment set up where aphids were placed on the paraffin film that served as a bridge between the *Parastagonospora nodorum* inoculated (SNB+) and non-inoculated (SNB−) leaves.

The influence of SNB pre-infection on the choice of aphids was assessed by exposing SNB− and SNB+ leaf segments to 11 adult wingless aphids per choice arena. This was done by placing one SNB+ and one SNB− wheat leaf segment of about 3.5 cm with the short cut edge close to each other in a Petri dish with 5% water agar. A piece of Parafilm of about 1.5 cm^2^ was placed on the agar, bridging the space between the two leaves (Figure 1). Eleven female aphids were released on the Parafilm bridge and then allowed to move freely between the inoculated and the non-inoculated leaf segments for 2 days. The movement and settlement of the aphid on the SNB+ or SNB− leaf was recorded 30 min, 3 h, 24 h and 48 h after the release of aphids.

In the first experiment, two wheat cultivars Zebra and Bjarne were used. The experiment had three replicates with 11 *R. padi* females for each replicate. Based on the results of the first experiment the second experiment was modified as follows: Only the cv. Bjarne was used and seven *R. padi* females were exposed to the choice situations for each replicate. The second experiment had five replicates. Since there was no difference on the choice and performance of the aphids between the two cultivars, we used one cultivar in the second experiment and increased the replication.

### 2.4. Statistical Analyses

Data on aphid preference and on SNB incidence (number of leaves infected per plant) and severity (percentage of leaves infected) were checked for normal distribution of data and subjected to statistical analysis using the software program Minitab [23]. The data with aphid number were log transformed before running the statistical test. The experiment on effect of aphid infestation of wheat plants on SNB development (severity and incidence) were conducted three times. Each experiment had 3–5 replications. The disease severity and incidence data were subjected to analysis of variance (ANOVA) with the general linear model (GLM) option of MINITAB, and the effect of the experiments, treatments and their interaction were determined. There was a significant variation among repeated experiments. In addition, there were a slight modification of the treatment combinations after experiment 1, and disease registrations intervals and number of disease assessments were slightly modified based on the disease development. So, the data from each experiment were analyzed and presented separately. Graphs were created in Sigma plot 13. There was no disease on control plants that was not inoculated with *P. nodorum*, and those plants were also free from aphids. Therefore, data from the uninoculated control plants were not analyzed statistically because disease severity and incidence values were zero.

## 3. Results

### 3.1. Effect of Aphid Infestation on SNB Development

Control plants (Aphid−, SNB−) were free of aphid and showed no symptom of SNB.

**Figure 2:**
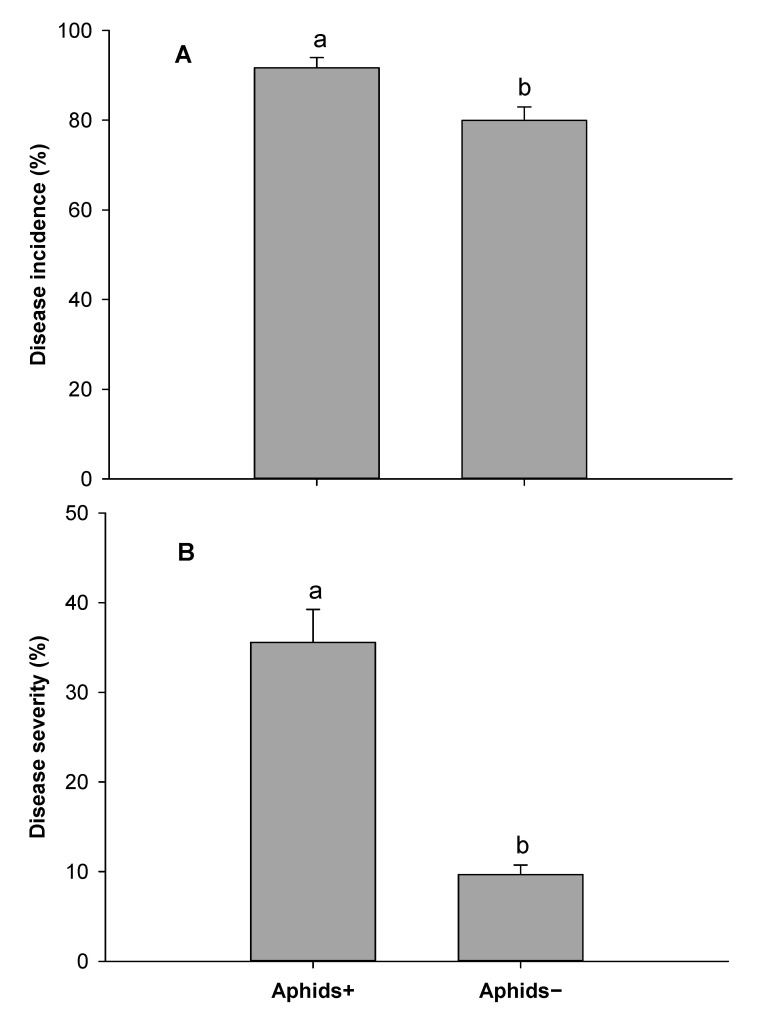
Stagonospora nodorum blotch disease 14 days after inoculation: Disease incidence (**A**) and severity (**B**) on aphid (*Rhopalosiphum padi)* infested (Aphids+) and non-infested (Aphids−) wheat plants from experiment 1. Error bars are standard error of the mean values and bars with different letters are different according Tukey’s test at *p* = 0.05.

**Figure 3:**
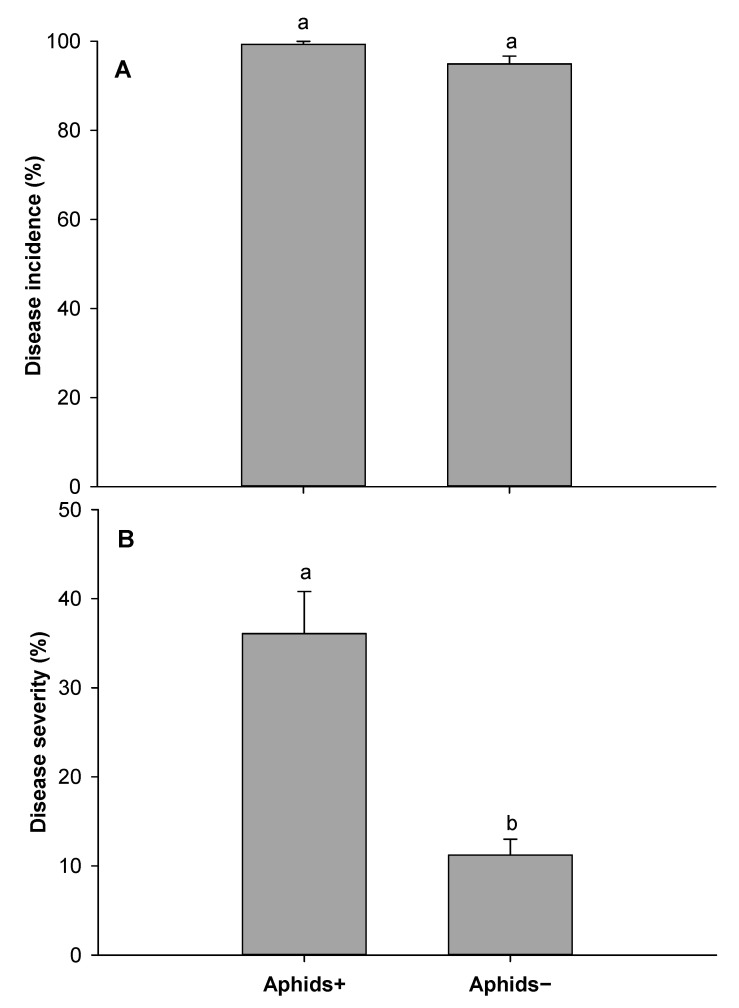
Stagonospora nodorum blotch 11 days after inoculation: Disease incidence (**A**) and disease severity (**B**) on aphid (*Rhopalosiphum padi*) infested (Aphids+) and non-infested (Aphids−) wheat plants from experiment 2. Error bars are standard error of the mean values and bars with different letters are different according Tukey’s test at *p* = 0.05.

**Figure 4:**
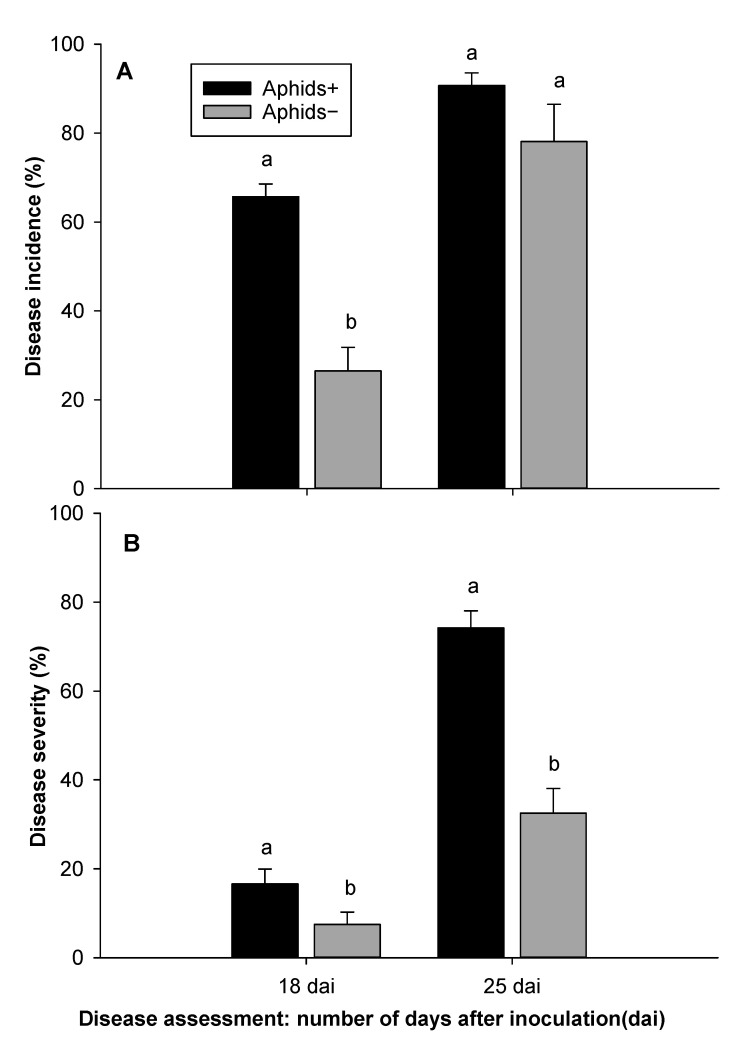
Stagonospora nodorum blotch 18 and 25 days after inoculation (dai): Disease incidence (**A**) and disease severity (**B**) on aphid (*Rhopalosiphum padi)* infested (Aphids+) and non-infested (Aphids−) plants from experiment 3. Error bars are standard error of the mean values and bars with different letters are different according Tukey’s test at *p* = 0.05.

SNB incidence was significantly higher on aphid-infested plants (Aphids+) than aphid-free plants (Aphids−) (F = 7.97, df = 1, 4, *p* = 0.048) for experiment 1 (Figure 2A). In experiment 2, there was no statistically significant difference in disease incidence (*p* = 0.06) (Figure 3A). In experiment 3, there was a significant difference in disease incidence between Aphids+ and Aphids− 18 days after inoculation assessment (F = 42.32, df = 1, 8, *p* = 0.001), but not significantly different 25 days after inoculation (Figure 4A). SNB severity was also significantly higher on aphid infested plants for experiment 1 (Figure 2B) (F = 9.38, df = 1, 4, *p* = 0.04), for experiment 2 (Figure 3B) (F = 24.14, df = 1, 6, *p* = 0.003) and for experiment 3 (Figure 4B) (F = 38.53, df = 1, 8, *p* < 0.001). Disease severity were about 4-fold, 3-fold and 2-fold in aphids infested plants compared with non-infested plants in experiments 1, 2 and 3, respectively.

### 3.2. Aphid Preference and Reproduction on P. nodorum Inoculated and Non-Inoculated Wheat Leaves

**Figure 5:**
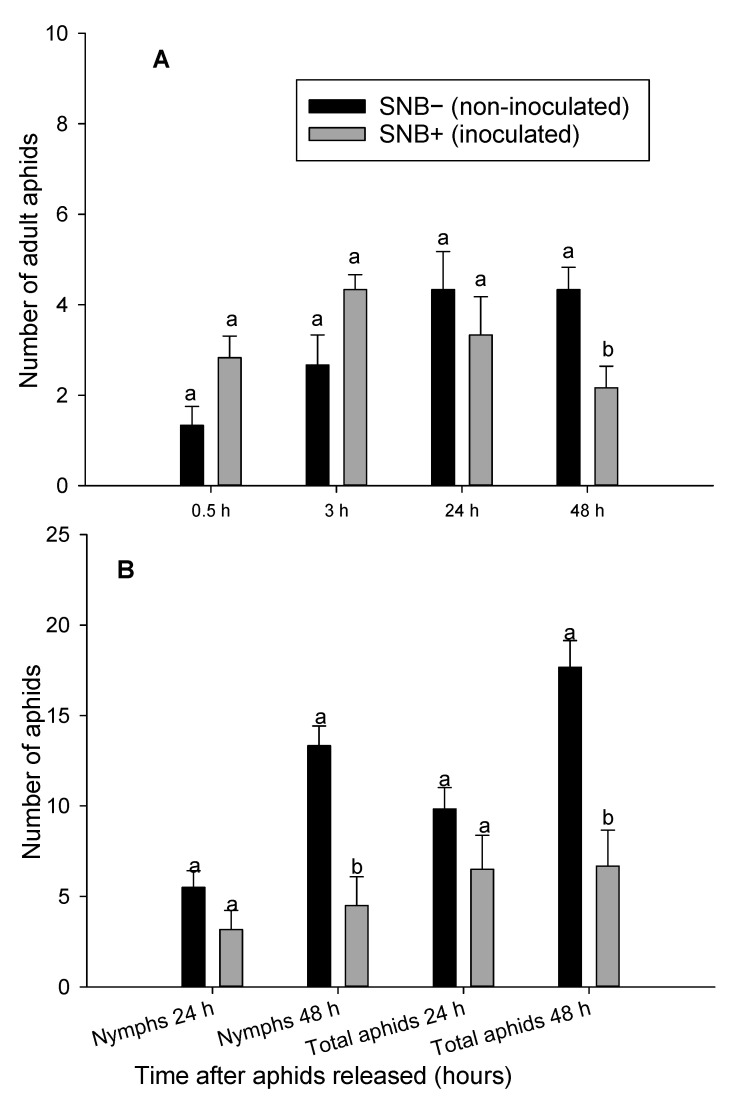
Number of adult aphids (*Rhopalosiphum padi)* (**A**) and number of adult aphids and nymphs (*Rhopalosiphum padi)* (**B**) on *Stagonospora nodorum* blotch inoculated (SNB+) and non-inoculated (SNB−) leaves of wheat 0.5, 3, 24 and 48 h after adult female aphid release. Results from experiment 1. Error bars are standard error of the mean values and bars with different letters within the same treatment group are statistically different at *p* = 0.05.

**Figure 6:**
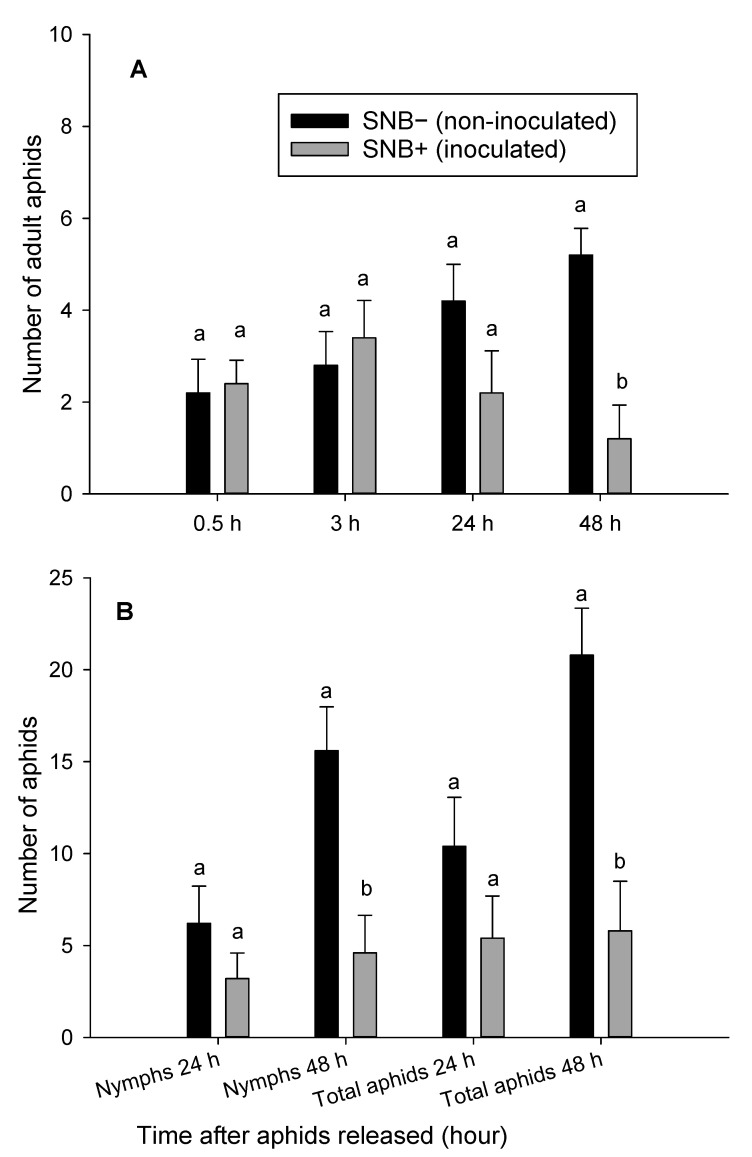
Number of adult aphids (*Rhopalosiphum padi*) (**A**) and number of adult aphids and nymphs (*Rhopalosiphum padi*) (**B**) on *Stagonospora nodorum* blotch inoculated (SNB+) and non-inoculated (SNB−) leaves of the wheat 0.5, 3, 24 and 48 h after adult female aphid release. Results from experiment 2. Error bars are standard error of the mean values and bars with different letters within the same treatment group are statistically different at *p* = 0.05.

In the preference (dual-choice) assay, significantly higher number of aphids moved and settled on non-inoculated (SNB−) leaves than on *P. nodorum* inoculated (SNB+) leaves (*p* ≤ 0.05) 48 h after aphids were released (Figure 5 and Figure 6). Adult aphids moved back and forth between the inoculated and non-inoculated leaves during the first 24 h, but after 48 h, significantly higher number of aphids moved, settled and started to produce progeny on non-inoculated leaves (SNB−) (Figure 5A and Figure 6A). In both experiments, the mean number of adult aphids and their progeny were significantly higher on the non-inoculated (SNB−) leaves than on inoculated leaves (SNB+) 48 h after release (Figure 5B and Figure 6B). The number of aphids were more than 2-fold on non-inoculated versus *P. nodorum* inoculated (SNB+) leaves 48 h after aphids were released (Figure 5B and Figure 6B).

## 4. Discussion

Our results show that the pre-infestation of wheat plants by Bird cherry-oat aphid (*R. padi*) predisposes the plants to *P. nodorum* and increases the severity and disease development of SNB. Further, our results show that *R. padi* thrives better on non-inoculated (SNB−) than inoculated (SNB+) wheat leaves.

Arthropods have been implicated in the epidemiology of several plant diseases [14]. Our studies agree with previous findings, which report that necrotrophic pathogen colonization is increased by prior tissue damaged by other pathogens or insects [8,9,10,11,12,13,14,15]. Honeydew from aphids are known to stimulate leaf senescence [3] and the combined effect of tissue damage by aphids and honeydew may predispose the plant to fungal disease. In wheat, aphid infestation predisposed plants to the necrotrophic fungus *F. graminearum*, and disease doubled on aphid infested plants [14]. Further, the large pine feeding aphids, *C. pinea*, increase the necrosis development and scleroderris canker of conifers caused by the fungus *Greimmeniella abietina* by providing infection courts, and the plants infested with aphids showed high disease severity caused by *G. abietina* (95% necrosis) compared to plants without aphids (50% necrosis) [15].

An exploitative colonization is a survival strategy among pathogens and pests that colonize a common host. The reduction in aphid preference and reproduction on SNB inoculated leaves compared to non-inoculated leaves could be due to poor nutrient availability and quality. Some aphid species are sensitive to nitrogen levels in leaves [24]. Necrotrophic pathogens reduce the nitrogen content of a leaf [13], although there are studies that show the opposite. One example of this is aphids feeding on bean leaves infected with the fungal pathogen faba-bean rust, *Uromyces viciae-fabae*, that leads to a rapid increase in aphid numbers. The components contributing for the increase in population are: Increase in mean relative growth rate (MRGR) by 25%, shorter maturation time by two days, increase in fecundity by 39% and increase in intrinsic rate (rm) by 48% of aphids feeding on the *U. viciae-fabae* infected leaves than on healthy leaves [13].

Other plant–pathogen–aphid interaction studies are in accordance with our results that necrotrophic pathogens negatively affect the choice of aphids between healthy and pathogen infected leaves [10,13]. In a tripartite interaction study that involved leaf beetles (*Gastrophysa viridula*), the rust fungus (*Uromyces rumicis*) and their host plant *Rumix obtusifolius,* the beetles were deterred by the rust infection [25]. Further, the biotrophic fungal wheat pathogen *Blumeria graminis* fsp.*tritici* reduced the fitness of the grain aphid (*Sitobion avenae*) by suppressing the feeding behavior, adult and nymph weight and fecundity and prolonged the developmental time [26]. *Parastagonospora nodorum* is a necrotrophic pathogen, and it may alter the cellular assimilate composition and phloem sap quality, which make the leaf unsuitable for aphid feeding and reproduction. SNB probably also change the leaf and glume surface structure, color and chemical composition, which may serve as a cue for the aphids. Aphids are known to employ a variety of sensory and behavioral mechanism to choose their preferred host tissue [27]. Although superficial cues such as epicuticular waxes [28], trichomes density, leaf surface texture and leaf color influences aphids’ behavior, performance and preference of their host tissue [27], the final discriminatory cue is after the aphid insert its stylet into the cells of the host. This suggests that intracellular substances or metabolites give aphids a reliable host selection cue [27]. It is known that plant pathogens induce changes in the intracellular substances in their host plants and that this can affect the performance and feeding behavior of insects [8,21].

Our findings that *R. padi* reproduce more on non-inoculated than *P. nodorum* inoculated leaves is in accordance with other plant, pathogen, herbivore interaction studies. For instance, on *B. cinerea*-infected broad bean plants, aphid performance and population growth parameters such as growth rate, fecundity, and intrinsic rate of natural increase were significantly inhibited and reduced [13]. Similarly, on rose plants Rosa hybrid cv. Sonia, aphid populations were significantly inhibited by pre-infection of the plants by *B. cinerea* [10]. In contrast, aphid performance was enhanced on *B. fabae*-infected faba bean plants [20]. An increased nutrient supply in *B. fabae* infected leaves were suggested to explain the increased performance of aphids [20]. In the tripartite interaction study mentioned above that involved leaf beetles (*G. viridula*), the rust fungus (*U. rumicis*) and their host plant *R. obtusifolius*, it was shown that if the female beetle oviposited on rust infected leaves it resulted in high larval mortality, low relative growth rate of the surviving larvae and reduced fecundity at the adult stage [25].

The success of multiple insects and pathogens colonizing the same host depends on their ability to compete with each other for the limited host tissue and their ability to breach host defenses. From this study we can suggest two types of plant (wheat)-aphid (*R. padi*)-pathogen (*P.nodorum*) interactions: (i) Pathogen (*P. nodorum*) modulated wheat-aphid (*R. padi*) interaction that has a negative effect on *R. padi* performance and reproduction; and (ii) aphid (*R. padi*) modulated wheat-pathogen (*P. nodorum*) interaction that has a positive effect on SNB development and spread.

## 5. Conclusions

Wheat plants are under constant attack by multiple pests and until now there are no studies on the interaction between the aphid *R. padi* and the SNB on wheat. Our findings indicate that prior infestation of wheat plants by aphids predisposed the plants to *P. nodorum* infection and colonization. We also found that SNB inoculated leaves do not attract aphids and they reproduce in a lower number on these leaves. These results are important to understand the interactions between multiple pests in wheat and hence how to develop new strategies in future integrated pest management (IPM). To be meaningful for integrated management of these pests, more detailed studies are needed on e.g., whether SNB inoculation of whole wheat plants may induce systemic resistance against aphids or not. Further, field experiments on how the time of infestation of aphids and time of aphids management options can affect the SNB on leaves and glum blotch development on wheat should be conducted.

## Figures and Tables

**Table 1:** Treatments and combinations in the experiment on interaction between *Rhopalosiphum padi* (cherry-oat aphid) and *Parastagonospora nodorum* (stagonospora nodorum blotch) on whole wheat plants.

Treatment Number ^1^	Treatment Combination	Abbreviation	Comments
1	*R. padi* infested, Insecticide sprayed, *P. nodorum* inoculated	Aphids+ SNB+	To evaluate the effect of aphid infestation on SNB development. Insecticide used to remove aphids before *P. nodorum* inoculation.
2	Water sprayed, *P. nodorum* inoculated	Aphids− SNB+	Positive control: To evaluate the effect of *P. nodorum* only
3	Insecticide sprayed, *P. nodorum* inoculated	Aphids− SNB+	Positive control: To evaluate if the insecticide affect *P. nodorum*
4	Untreated control	Aphids− SNB−	Negative control: To control for contamination of clean plants with *R. padi* or *P. nodorum*

^1^ There was no significant difference between water-sprayed and insecticide-sprayed plants on SNB development, so the data from treatment 2 and 3 were pooled and results presented as ‘Aphids−’.

**Table 2:** Dates of wheat seed sowing, aphid release, insecticide application, inoculation of *P. nodorum* and total number of leaves included for disease incidence assessment on the different experiments.

	Experiment 1	Experiment 2	Experiment 3
Date of sowing	06.09.2013	09.01.2014	08.09.2014
Date of aphid release	14.10.2013	20.02.2014	13.10.2014
Insecticide removal of aphids	21.10.2013	28.02.2014	23.10.2014
*P. nodorum* inoculation	22.10.2013	03.03.2014	24.10.2014
Number of replications	3	4	5
Number of leaves assessed for SNB incidence	409	585	702
Disease assessment dates ^1^	05.11.2013	13 & 20.03.2014	04, 11, & 18.11.2014

^1^ Disease assessment was discontinued when the disease incidence reached 100%.

## Data Availability

The data presented in this study are available on request from the corresponding author.
